# Determining amino acid requirements in humans

**DOI:** 10.3389/fnut.2024.1400719

**Published:** 2024-07-18

**Authors:** Alyssa Paoletti, Glenda Courtney-Martin, Rajavel Elango

**Affiliations:** ^1^Research Institute, Hospital for Sick Children, Toronto, ON, Canada; ^2^Department of Nutritional Sciences, University of Toronto, Toronto, ON, Canada; ^3^Department of Kinesiology, University of Toronto, Toronto, ON, Canada; ^4^Department of Pediatrics, University of British Columbia, Vancouver, BC, Canada; ^5^School of Population and Public Health, University of British Columbia, Vancouver, BC, Canada; ^6^BC Children’s Hospital Research Institute, BC Children’s Hospital, Vancouver, BC, Canada

**Keywords:** amino acids, requirements, humans, IAAO, stable isotope

## Abstract

Amino acids form the building blocks of body protein. Dietary protein sources provide the amino acids needed, but protein sources vary widely in amio acid composition. To ensure humans can meet body demands for amino acids, amino acid intake recommendations are provided by the Dietary Reference Intakes (DRI) and by Food and Agriculture Organization/World Health Organization/United Nations University (FAO/WHO/UNU). Current amino acid intake recommendations, however, are based on data collected predominantly from young adult males. The development of the minimally invasive indicator amino acid oxidation (IAAO) method has permitted the evaluation of amino acid requirements in various vulnerable populations. The purpose of this review is to discuss recent amino acid requirement studies in school-age children, pregnant females and the elderly determined using the IAAO technique. These requirements will help to inform evidence-based recommendations that will help to guide dietary guidelines.

## Introduction

In the human body, protein is the chief functional and structural constituent in every cell (1). During development, dietary protein is necessary for growth plus maintenance and for maintenance alone during all other stages of life. The most important nutritional aspect of dietary protein are the consituent amino acids. Among the 20 amino acids that constitute human body protein 9 are indispensable (histidine, isoleucine, leucine, lysine, methionine, phenylalanine, threonine, tryptophan, valine), which means they cannot be made in the body and must be supplied in the diet (1). There are five dispensable amino acids (alanine, aspartic acid, asparagine, glutamic acid, serine) that can be synthesized in the body whereas the remaining six are conditionally indispensable (arginine, cysteine, glutamine, glycine, proline, tyrosine) meaning that they can be made by the body however their synthesis becomes limiting under specific conditions.

The Food and Agriculture Organization (FAO) have acknowledged that indispensable amino acids should be treated as individual nutrients since the amino acid composition of foods vary greatly and *in vivo* amino acids have various regulatory roles (i.e., precursors for coenzymes, hormones, nucleic acids and other molecules) (2). In addition, the nutritional value of dietary protein is determined by the most limiting indispensable amino acid in foods. Therefore, dietary protein sources are categorized as either high- or low-quality. The classification is determined by the amino acid score of the food which is the amount of amino acid supplied by the food relative to their corresponding amino acid requirements. Animal protein sources (e.g., eggs, meat, fish) provide all indispensable amino acids in quantities and ratios adequate to meet human requirements, whereas many indispensable amino acids in plant foods occur in quantities and ratios that may not meet requirements under all conditions (3). Therefore, in a normal sized meal, plant proteins may fail to fulfil an indispensable amino acid requirement, which is referred to as the limiting amino acid (4). For individual’s adhering to a strict plant-based diet, the limiting amino acid will limit the body’s capacity to make proteins (3). This is because when an indispensable amino acid is deficient in the diet, all other amino acids, appear in relative excess and will be oxidized since there is no substantial storage of amino acids in the body (5). Over the long term, this may lead to negative consequences on whole-body protein metabolism. Thus, as a first step we need knowledge of amino acid requirements across life-stage groups to understand how to meet their needs with different dietary protein sources. This is especially relevant in today’s landscape where plant-based diets are encouraged and increasingly popular.

Current dietary amino acid intake recommendations are outlined in the Dietary Reference Intakes (DRI) issued by the National Academy of Science, Engineering and Medicine [NASEM, formerly—Institute of Medicine (IOM)] (1, 6). Global recommendations were provided by the Food and Agriculture Organization/World Health Organization/United Nations University (FAO/WHO/UNU), and are stated as mean and safe intake levels of amino acids, whereas the DRI uses an estimated average requirement (EAR) and recommended dietary allowance (RDA) (3, 6). However, the mean intake is equivalent to the EAR and the value is set to meet requirements for half (50%) of a healthy population. Similarly, the safe intake level and the RDA are the same and aim to meet the requirements of 97 to 98% of a healthy population. Both the DRI and FAO/WHO/UNU recommendations are provided for adults (19y+) based on values determined in studies conducted in young adults. For all other age groups, including children, pregnant and lactating people a factorial method with maintenance needs from adult data with growth estimates calculated were used to set recommendations. Clearly, data on other populations is lacking and therefore, must be determined directly with a sense of urgency.

## Evolution of methods to determine amino acid requirements

Determination of amino acid requirements requires graded levels of the test amino acid above and below the expected requirement to be fed to participants while measuring a definable and relevant biological outcome (7). Currently, the outcome of all existing methods is a surrogate measure of protein synthesis (8). Traditionally, amino acid requirements were determined using nitrogen balance studies (9–15)—measuring nitrogen intake and excretion. Briefly, as the test amino acid intake increases there is a progressive increase from negative to zero-nitrogen balance until the requirement is reached. The limitations of nitrogen balance are well described and include the limited range of test amino acids studied, its cumbersome nature requiring precise measurements of balance among several other isssues (16). Readers are referred to reviews for an extensive background on the limitations of nitrogen balance (16, 17). The lack of amino acid requirement studies in vulnerable groups such as infants, pregnancy and elderly are attributed to these limitations.

As reviewed by Pencharz et al., the advent of carbon oxidation methods using ^13^C-labelled amino acids—including direct amino acid oxidation (DAAO), indicator amino acid oxidation (IAAO) and 24 h IAAO have allowed for the determination of amino acid requirements in various vulnerable populations (7). Briefly, for DAAO, when the amino acid is fed below the requirement there is no change in oxidation until the requirement is met, after which there is an increase in the oxidation. For the IAAO and 24 h IAAO, the oxidation of an indicator amino acid (another indispensable amino acid) response falls as the test amino acid intake increases, until the requirement is reached after which there is no further change in oxidation (5, 7). The 24 h approach is in essence an adaption of the 8 h, fed state IAAO protocol to include both the fed/fasted states as Indicator Amino Acid Balance (IAAB). According to the FAO/WHO/UNU (2007) report, *“…on theoretical grounds the most reliable approaches are the 24 h indicator/carbon balance approaches.”* As previously reviewed (18) similar amino acid requirement estimates have been derived using both 24 h-IAAB and 8 h fed state IAAO methods with no systematic difference in estimates. Moreover, within the 24 h IAAO studies, when requirements are compared between the 12 h fed and 8 h fed state, there is no difference in requirement estimates (19, 20). Prior adaptation to test amino acid intake to 8 h, 2d, or 6d also did not significantly affect IAAO for lysine (21) or threonine requirements (22). Clearly, the 8 h-IAAO is advantageous in studying AA requirements due its minimal invasiveness including a single day of adaption to the test amino acid (22), oral isotope administration with meals (23), and measurement of ^13^CO_2_ in breath (23). Given these advantages, the method has been successfully applied to study amino acid requirements in understudied groups like children, elderly and in patients with disease (24–27). As a result, new datasets are emerging on amino acid requirements. The following sections will outline recent IAAO-derived amino acid requirements determined in healthy children, children with disease, pregnancy and elderly groups.

## Amino acid requirements in healthy children and children with disease

A comprehensive list of the IAAO-derived amino acid requirement studies in healthy and children with certain conditions are illustrated in Table 1. Healthy children aged 6–10y have similar amino acid requirements compared to adults for total branched-chain amino acids (BCAA, isoleucine+leucine+valine), lysine, total sulfur amino acids (TSAA, methionine+cysteine) and tryptophan, suggesting maintenance needs are the same, considering the fact that the 8 h-IAAO protocol are short-term studies (25). However, in the case of different disease state states amino acid requirements are changed.

**Table 1:** Amino acid requirements in healthy children and under certain conditions determined using the IAAO method.

Population	Amino Acid (mg/kg/d)^a^					
	Total BCAA	Methionine (no cysteine)	Methionine + Cysteine	Lysine	Tryptophan	Phenylalanine
Healthy North American children	147 (28)	12.9 (29)	5.8 (30)	35 (31)	4.7 (32)	–
Healthy North American adults	144 (33)	12.6 (34)	4.5 (35)	36 (36)	4 (37)	9.1 (38)
Cholestatic liver disease children	209 (39)	–	–	–	–	–
Post-liver transplant children	172 (40)	–	–	–	–	–
Maple syrup urine disease	45 (41)	–	–	–	–	–
Chronic renal insufficiency children	–	12.6 (42)	7.3 (42)	–	–	–
Healthy Indian children	–	–	–	33.5 (43)	–	–
Stunted Indian children (with gut parasites)	–	–	–	42.8 (44)	–	–
Stunted Indian children (after treatment of parasites)	–	–	–	35.5 (44)	–	–
Children with phenylketonuria (PKU)	–	–	–	–	–	14 (45)

a Values described are mean amino acid requirements.

Children with liver disease have ~40% increased total BCAA requirements (39). Whereas, post liver transplant, in the same group of children, the requirement is increased by ~17% (39). In patients with maple syrup urine disease, the requirement for total BCAA is much lower because the demand for branched chain amino acids is low, due to BCAA catabolic enzyme defect (41). Thus, this was the first study to estimate a minimum total BCAA needs which are ~69% lower compared to healthy children. The TSAA requirements in children with chronic renal insufficiency are the same as healthy children (42). Yet, the demand for obligatory methionine appears to increase by ~25% in this group relative to healthy controls (42). Healthy Indian children have similar lysine needs as healthy Canadian children (43, 46) while, the lysine requirement is increased ~21% by the presence of gut parasites in under-nourished Indian children (44). Thus, amino acid needs vary depending on the type and severity of disease, and the findings described above lead the way to revising dietary guidelines for disease management.

## Amino acid requirements in pregnancy

Amino acid requirements during human pregnancy has been infrequently studied due to the invasive nature of the nitrogen balance method. Due to the minimally invasive nature of the IAAO method, a series of studies across two distinct stages of pregnancy—early (~16 wk) and late (~36 wk) gestation have been conducted (Table 2). The mean protein needs in early-stage pregnancy is 1.2 g/kg/d (47), and increased compared to mean protein needs (0.9 g/kg/d) determined in young males (52). During late stages of pregnancy protein needs increase further to 1.52 g/kg/d (47). However, amino acid requirements do not increase proportionally, compared to protein needs. The findings suggest that while protein needs increase in late stage, not each individual amino acid requirement follows the same pattern. Lysine, and TSAA requirements during early pregnancy stages are similar to non-pregnant needs, however phenylalanine needs (in the presence of tyrosine) increase by 66% compared to non-pregnant needs, as well as the total aromatic amino acid (TAA, phenylalanine in the absence of tyrosine) requirements (49). All determined amino acid requirements (lysine, TSAA, TAA and phenylalanine) increase by late stages of pregnancy (48, 49, 51), albeit at different amounts. Most interestingly, glycine a conditionally indispensable amino acid was shown to be indispensable in human pregnancy by late stages of pregnancy (50, 53). It is of importance to note that in the glycine in pregnancy study, the amount of protein was fed at current pregnancy protein needs (0.88 g/kg/d), which further validates the finding that current protein intake recommendations in pregnancy are underestimates. Further work is required to complete the remaining indispensable amino acid requirements in different phases of pregnancy.

**Table 2:** Amino acid requirements in early and late-stage pregnancy determined using the IAAO method.

Nutrient^a^	Non-pregnant needs	Early-stage pregnancy (~16 weeks)	Late-stage pregnancy (~36 weeks)	Reference
Protein (g/kg/d)	0.9	1.2	1.52	(47)
Lysine (mg/kg/d)	36	37	50	(48)
Phenylalanine (mg/kg/d)	9	15	21	(49)
Phenylalanine+tyrosine	42	44	50	(49)
Glycine (mg/kg/d)	–	–	40	(50)
Methionine+cysteine (mg/kg/d)	13	11	17	(51)

a Values described are mean amino acid/protein requirements.

## Amino acid requirements in healthy adults >60 years

Amino acid requirements in elderly have also been infrequently studied due to methodoligcal invasiveness. With the global population now aging, there is an increased need to determine amino acid needs in elderly. Table 3 provides a complete list of all amino acid requirements done in healthy males and females >60 years of age. Similar protein needs have been determined for young and older adults using the IAAO-method, although the determined values are higher than current mean recommendations of 0.66 g/kg/d (52, 54, 55). Amino acid requirements however are not proportionally the same compared to young adults, and are influenced by sex. Phenylalanine requirements were also found to be the same in elderly males and females as healthy young adult male requirement (38, 57). However, leucine requirements in elderly males and females was found to be nearly double that of healthy young males (58, 61) suggesting that while needs for total nitrogen is unchanged with age, there are increased demands for specific amino acids. More recently, the TSAA requirement was affected by sex, with older males having a higher requirement compared to older females and healthy young males (34, 59). Interestingly, the minimum methionine (in the presence of adequate cysteine) requirement was the same between sexes and healthy young adult males (35, 60). These series of amino acid requirement studies highlights the need to assess requirements between sexes. Additionally, the existing studies conducted in older adults include subjects aged 60–69 y old with few aged 70–79 and >80 y old. It has been shown that there is ~5% decrease in whole-body protein turnover when stratified by decade of life with aging (62). As a result, further studies are necessary to experimentally derive the amino acid requirements for these advanced age groups.

**Table 3:** Amino acid requirements in elderly males and females >60 years of age determined using the IAAO method.

Nutrient^a^	Young males	Elderly males (>60 years old)	Elderly females (>60 years old)	Reference
Protein (g/kg/d)	0.93	0.94	0.96	(54–56)
Phenylalanine (mg/kg/d)	9.1	9.3	8.4	(57)
Leucine (mg/kg/d)	39	77.8	78.2	(58)
Methionine (mg/kg/d)	13	26.2	17.1	(59)
Methionine + Cysteine (mg/kg/d)	4.5	5.4	4.6	(60)

a Values described are mean amino acid/protein requirements.

## Perspective: amino acid requirements and dietary patterns

As summarized above, amino acid requirements determined across a wide range of physiological stages and disease conditions vary based on several factors, and that a factorial method may not be adequate to give amino acid intake recommendations. A few key points must be discussed here with respect to the fact that the experiments to determine amino acid requirements are conducted with adequate energy, following an ideal amino acid composition (egg protein pattern) using a highly bioavailable source (crystalline amino acids). Thus, the determined values represent a true ‘minimum’ amino acid requirement. Humans consume foods following different dietary patterns – omnivorous, vegetarian, vegan diets etc., and will influence the minimum amino acid requirements. Specifically, following a strict vegan diet would rely on plant-based sources of protein, which would have lower digestibility, lower dietary calorie density and likely a less ideal pattern of all the indispensable amino acids. In theory, amino acid intakes would need to be higher in these instances to meet body amino acid needs. Furthermore, additional nutritional needs and demands would be different based on physiology, for example actively growing children, pregnant females would have increased energy needs, while elderly sedentary individuals would have lower energy needs. In addition, living in poor socio-economic and living conditions might increase the needs for some amino acids, as shown by our lysine requirement study in under-nourished children with active parasitic infection. Thus, translation of our amino acid requirement values to dietary guidelines needs to consider several factors. It is also important to note here that conceptually DRI and FAO/WHO/UNU recommendations as defined by the EAR and RDA for all nutrients are a ‘minimum’ and not a ‘maximum’, that ensures populations can consume diets to maintain health and quality of life.

## Summary and conclusions

Current amino acid intake recommendations have been determined based on studies conducted in young adult males. For all other life stages a factorial approach was used, primarily due to lack of data. The minimally invasive IAAO method has been successfully applied in vulnerable populations and in different disease states in children. New datasets are also developing for pregnancy and for the elderly population so that we can provide and inform evidence-based recommendations. These datasets are urgently needed since amino acid needs vary based on disease condition, across pregnancy stages and between sexes in the elderly population. At the same time, several key life stages such as adolescents, young female adults and lactation amino acid requirements remain to be investigated. The advent of plant-based diets warrants the need to determine indispensable amino acid requirements with a sense of urgency to appropriately provide nutritional guidelines and recommendations on how to meet individual needs.

## Author contributions

AP: Conceptualization, Writing – original draft, Writing – review & editing. GC-M: Conceptualization, Supervision, Writing – review & editing. RE: Conceptualization, Funding acquisition, Writing – review & editing.
